# The pharmacokinetic and safety profile of single-dose deferiprone in subjects with sickle cell disease

**DOI:** 10.1007/s00277-021-04728-0

**Published:** 2022-01-04

**Authors:** Denis Soulières, Jules Mercier-Ross, Caroline Fradette, Anna Rozova, Yu Chung Tsang, Fernando Tricta

**Affiliations:** 1 Centre Hospitalier de l’Université de Montréal (CHUM), Montreal, QC Canada; 2 Chiesi Canada Corporation, Toronto, ON Canada; 3 Apotex Inc., Toronto, ON Canada; 4 Faculty of Pharmacy, University of Toronto, Toronto, ON Canada

**Keywords:** Sickle cell disease, Iron chelation, Deferiprone, Pharmacokinetics

## Abstract

Patients with sickle cell disease (SCD) who undergo repeated blood transfusions often develop iron overload. Deferiprone (Ferriprox®) is an oral iron chelator indicated for the treatment of transfusional iron overload due to thalassemia syndromes and has been recently approved as a treatment for iron overload in adult and pediatric patients with SCD and other anemias. The present study aims to characterize the pharmacokinetic (PK) profile of deferiprone (DFP) in adult subjects with SCD. In this phase I, open-label study, subjects with SCD were administered a single 1500 mg dose of DFP. Blood and urine samples were collected for PK assessments of DFP and its main metabolite, deferiprone 3-O-glucuronide (DFP-G). Eight subjects were enrolled and completed the study. Following drug administration, serum levels of DFP and DFP-G rose to maximum concentrations at 1.0 and 2.8 h post-dose, respectively. The half-lives of DFP and DFP-G were 1.5 and 1.6 h, respectively. The majority of administered drug was metabolized and excreted as DFP-G, with less than 4% excreted unchanged in urine up to 10 h post-dose. Subjects received a safety assessment 7 (± 3) days post-dose. Two subjects reported mild adverse events unrelated to the study drug, and no other safety concerns were reported. The PK profile of DFP in SCD subjects is consistent with previous reports in healthy adult volunteers, suggesting no special dosing adjustments are indicated for this population. These findings provide valuable insight for treating iron overload in patients with SCD, who have limited chelation therapy treatment options (trial registration number: NCT01835496, date of registration: April 19, 2013).

**Supplementary Information:**

The online version contains supplementary material available at 10.1007/s00277-021-04728-0.

## Introduction

Sickle cell disease (SCD), an inherited disorder that reduces the capacity of red blood cells to carry oxygen throughout the body, affects approximately 100,000 people in the USA, and an estimated 300,000–400,000 children are born with SCD world-wide each year [1–3]. According to data from the Globin Research Network for Data and Discovery (GRNDaD) Registry, 20.4% of adults with the HbSS form of SCD rely on chronic blood transfusions as part of their disease management [4]. Because the body has no physiological mechanism to actively excrete excess iron, many of these patients develop transfusional iron overload [5]. Repeated blood transfusions can lead to increased iron burden, indicated by high serum ferritin levels (> 1000 ng/mL) and greater than 50% of saturated transferrin [6–8], resulting in iron buildup in the liver, heart, and occasionally endocrine organs [9, 10]. Patients with SCD and transfusional iron overload have a higher incidence of acute painful crises (64% vs 38%), organ failure (71% vs 19%), and mortality (64% vs 5%) [7] compared with patients with SCD and normal iron levels. Iron chelation therapy is necessary to manage transfusional iron overload, but some iron chelation treatments, such as deferasirox, may cause renal and hepatic failure [5, 11]. Approximately 40% of patients included in the GRNDaD registry have proteinuria or albuminuria, a comorbid indicator of renal impairment, and these patients are significantly limited in iron chelation treatment options [4, 5, 12]. While renal impairment is fairly common in patients with SCD, several other complications such as liver disease, infections, cardiovascular events, and malignancies are also highly correlated with transfusional iron overload and contribute to morbidity and mortality [7, 8, 13]. The comorbidities associated with SCD highlight an unmet need in the therapeutic landscape for an iron chelator that is safe in this patient population.

Deferiprone (Ferriprox®) is an oral iron chelator indicated for treatment of iron overload in transfusion-dependent patients with thalassemia syndromes [14]. Its use is not associated with renal toxicity and is well tolerated in patients with decreased renal function [14, 15]. Furthermore, systemic exposure to deferiprone (DFP) was not altered in subjects with renal impairment, and no dose adjustment was required [15]. Deferiprone was recently approved by the US Food and Drug Administration as a first-line therapy for the treatment of iron overload in pediatric and adult patients with SCD or other transfusion-dependent anemias [14].

In previous studies [14], the pharmacokinetic (PK) profile of a single 1500 mg oral dose of DFP immediate release (IR) tablets was characterized in healthy volunteers. The mean maximum measured serum concentration (*C*_max_) for DFP was 20.0 µg/mL, area under the serum concentration versus time curve to infinity (AUC_0-inf_) was 50 µg*h/mL, and time to reach the *C*_max_ (*T*_max_) was approximately 1–2 h. The elimination half-life of deferiprone was reported to be approximately 2 h. However, the PK profile of DFP and its main metabolite, deferiprone 3-O-glucoronide (DFP-G), has not been described in those with SCD. Thus, the objective of the present study was to characterize the single-dose PK and safety profiles of DFP in subjects with SCD and to evaluate whether SCD causes alterations in the metabolism and excretion of DFP that necessitate dosing adjustments in this population.

## Methods

### Study design and treatment

This was a phase I, single-center, nonrandomized, open-label, single-dose study. Eligible subjects visited the study center to undergo baseline procedures (see Online Resource 1) and returned early the next morning in a fasted state to undergo pre-dosing procedures (see Online Resource 1). Subjects were administered a single 1500 mg oral dose of DFP (three 500 mg IR tablets). Previous PK studies conducted in healthy volunteers employed a single standardized dose of 1500 mg of DFP; thus, the same dose was used in the current study in order to compare the PK results of adults with SCD with those of healthy adults. Blood samples for serum PK assessments were collected pre-dose and at 0.25, 0.50, 0.75, 1, 1.33, 1.66, 2, 2.5, 3, 4, 6, 8, and 10 h post-dose. Urine samples to assess PK parameters were collected −2 to 0 h pre-dose, and 0 to 2, 2 to 4, 4 to 6, and 6 to –10 h post-dose.

### Study population

Study participants were males and females aged 18–45 years who had a primary diagnosis of SCD confirmed by high-performance liquid chromatography (HPLC); a body weight of ≥ 50 kg and body mass index (BMI) between 18 and 32 kg/m^2^; absolute neutrophil count (ANC) of > 1.5 × 10^9^/L; and confirmation of effective contraception (and partner) 14 days prior to dosing and for 30 days afterwards. Exclusion criteria included a history of hypersensitivity to DFP; use of DFP within the last 3 months; history of malignancy; evidence of abnormal liver function (ALT > 5 times the upper limit of normal or creatinine levels > 2 times upper limit of normal); a serious or unstable illness within the past 3 months; hemodialysis during the week prior to dosing or planned for the day of dosing; difficulty providing blood samples; disorders or surgery of the gastrointestinal tract that might interfere with drug absorption or otherwise influence the PK; clinically significant abnormalities on 12-lead electrocardiogram (ECG); use of tobacco/nicotine-containing products for at least 3 months prior to study drug administration; use of any drugs metabolized by the UGT1A6 enzyme [16, 17] within the past 14 days; treated with an investigational drug within the past 30 days; or were pregnant or nursing.

### Outcomes and assessments

The PK parameters assessed for DFP and DFP-G were *C*_max_, *T*_max_, area under the serum concentration versus time curve to the last measurable concentration (AUC_0-t_), AUC0-inf,﻿ apparent terminal elimination half-life (*T*½), total body clearance corrected for bioavailability (CL/F; measured for DFP only), renal clearance (CLr), amount excreted in urine (A_e_), and fraction of dose excreted in urine (*F*_e_).

Safety assessments included adverse event monitoring, clinical laboratory test results, vital sign measurements, 12-lead ECG, and use of concomitant medications. Participants returned to the study site at 7 ± 3 days post-dose for safety information and to provide a blood sample for measurement of ANC to ensure patients were not at risk for developing DFP-induced neutropenia or agranulocytosis [14].

### Statistical methods and analysis

Serum and urine samples were assayed for DFP and DFP-G using a validated high-performance liquid chromatography with mass spectrometric detection method (HPLC–MS/MS). PK analyses were conducted using the validated software Phoenix® WinNonlin® Version 6.3 (for serum) and SAS® Version 9.3 (for urine). The PK parameter values for DFP and DFP-G were derived from individual serum concentration time and urinary excretion profiles and summarized using descriptive statistics (arithmetic mean, standard deviation, median, minimum, and maximum). Safety data for continuous variables were summarized using descriptive statistics, and discrete variables were tabulated with frequency tables. The PK population analysis included all subjects who had sufficient PK data to derive the value of at least one PK parameter. The safety population included all subjects who received DFP.

## Results

### Subject demographics and medical history

Demographics are shown in Table 1. Eight subjects (5 females; 3 males) with a primary diagnosis of sickle cell disease (HbSS genotype) and mean (SD) age of 33.0 (5.9) years were enrolled and completed the study. All subjects were Black (Table 1). A summary of study subjects’ medical history and relevant clinical laboratory results are shown in Table 2. Notably, one subject was glucose-6-phosphate dehydrogenase–deficient. None of the study subjects had total bilirubin levels above 4XN [18, 19], and no subjects had a significant increase in any other hemolysis biomarkers prior to DFP administration (Table 2). Only one subject had frank proteinuria and microalbuminuria. A second subject had mild microalbuminuria at study inclusion that was isolated and not found to be persistent on follow-up.

**Table 1:** Demographic characteristics

Demographic variable	Overall *N* = 8
Age (years) Mean (SD) Median (minimum, maximum)	33.0 (5.9) 35.0 (25, 40)
Sex (*n* [%]) Male Female	3 (37.5%) 5 (62.5%)
Race (*n* [%]) Black	8 (100%)
Ethnicity African Non-Hispanic/Latino	1 (12.5%) 7 (87.5%)

*SD* standard deviation

**Table 2:** Summary of subject medical history and relevant biochemistry evaluations

Patient ID	Weight (kg)	Serum biochemistry				Urinalysis	Medical history
		Hg (g/L)	Serum ferritin (μg/L)	Bilirubin (total)^a^ μmol/L	Creatinine^b^ μmol/L	Urine protein/creatinine ratio^b^ N = normal; A = abnormal	
1	68.4	117	610	55	69	A	Sickle cell anemia (HbSS); cholecystectomy; osteopenia; retinal degeneration; priapism
2	85.9	106	373	27	42	N	Sickle cell anemia (HbSS); splenectomy; retinopathy; allergy to morphine; pulmonary hypertension; asthma
3	44.0	94	1904	51	36	N	Sickle cell anemia (HbSS); uterine fibroids; systolic murmur; pulmonary hypertension; dysmenorrhea; cerebrovascular accident; cholecystectomy; splenectomy
4	66.7	69	25	20	42	N	Sickle cell anemia (HbSS); eczema; back pain; dysmenorrhea; arthralgia; major depression; cholecystectomy; amygdalectomy
5	62.6	110	1975	27	77	N	Sickle cell anemia (HbSS); pulmonary hypertension; glucose-6-phosphate dehydrogenase deficiency; inguinal hernia repair
6	69.5	93	2244	79	56	N	Sickle cell anemia (HbSS); orthopedic procedure; osteonecrosis; gastroesophageal reflux; depression
7	67.0	79	65	90	61	N	Sickle cell anemia (HbSS); cholecystectomy; microalbuminuria
8	64.8	113	1198	77	50	N	Sickle cell anemia (HbSS); cholecystectomy; ruptured aneurysm

^a^Biomarker for hemolysis; ^b^biomarker for hyperfiltration. *Hg* hemoglobin

### Serum PK

**Fig. 1:**
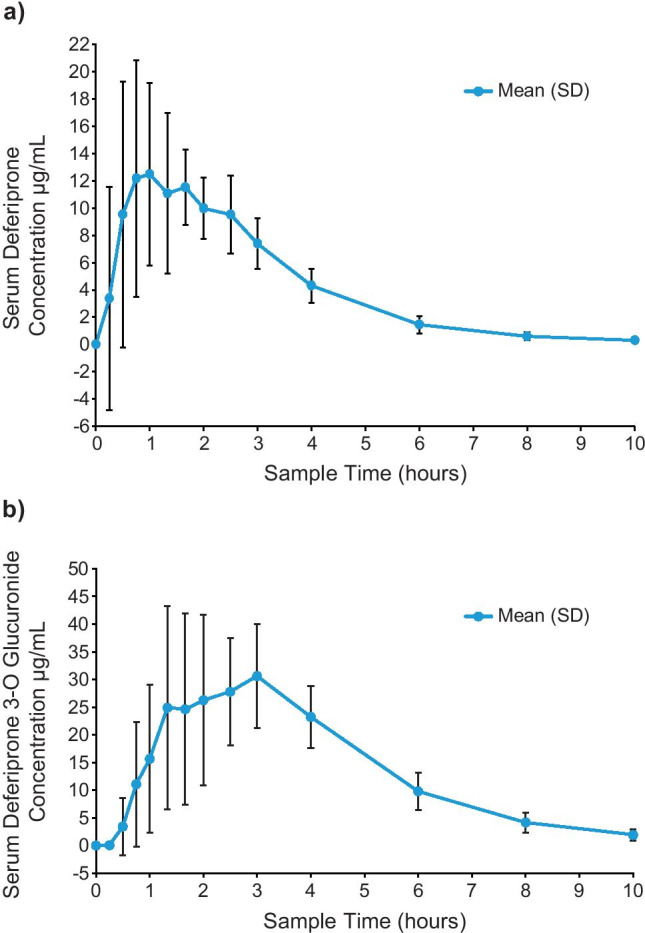
**a** Serum concentrations of deferiprone and **b** deferiprone 3-O-glucuronide following a 1500 mg dose of deferiprone

Following a single 1500 mg oral dose of DFP, the mean (SD) *C*_max_ for DFP was 17.6 (5.8) µg/mL, median *T*_max_ was 1.0 h post-dose, mean (SD) serum AUC_0-inf_ was 43.4 (5.4) µg*h/mL, half-life was 1.5 (0.2) h, and mean (SD) CL/F was 35.1 (4.5) L/h. For DFP-G, the mean (SD) *C*_max_ was 33.0 (11.8) µg/mL, median *T*_max_ was 2.8 h, mean (SD) serum AUC_0-inf_ was 142.7 (47.0) µg*h/mL, and half-life was 1.6 (0.2) h (Table 3). Mean (SD) DFP serum concentrations across the 10-h sampling period are shown in Fig. 1a. Mean (SD) DFP-G serum concentrations across the 10-h sampling period are shown in Fig. 1b.

**Table 3:** Summary of serum deferiprone (DFP) and deferiprone 3-O-glucuronide (DFP-G) pharmacokinetic parameters

Parameter	DFP	DFP-G
	Mean ± SD	Mean ± SD
*C*_max_ (µg/mL)	17.6 ± 5.8	33.0 ± 11.8
*T*_max_ (h)	1.0 (0.5, 2.5)^a^	2.8 (1.3, 3.0)^a^
AUC_0-t_ (µg*h/mL)	42.7 ± 5.3	138.1 ± 46.1
AUC_0-inf_ (µg*h/mL)	43.4 ± 5.4	142.7 ± 47.0
*T*_1/2_ (h)	1.5 ± 0.2	1.6 ± 0.2
CL/F (L/h)	35.1 ± 4.5	NC

^a^*T*_max_ median (minimum, maximum)

*AUC*_*0-Iinf*_ area under the serum concentration vs time curve to infinity; *AUC*_*0-t*_ area under the serum concentration vs time curve to the last measurable concentration; *CL/F* total body clearance corrected for bioavailability; *C*_*max*_ maximum measured serum concentration; *NC* not calculated; *T*_*½*_ apparent terminal elimination half-life; *T*_*max*_ time to reach the maximum serum concentration

### Urine PK

**Fig. 2:**
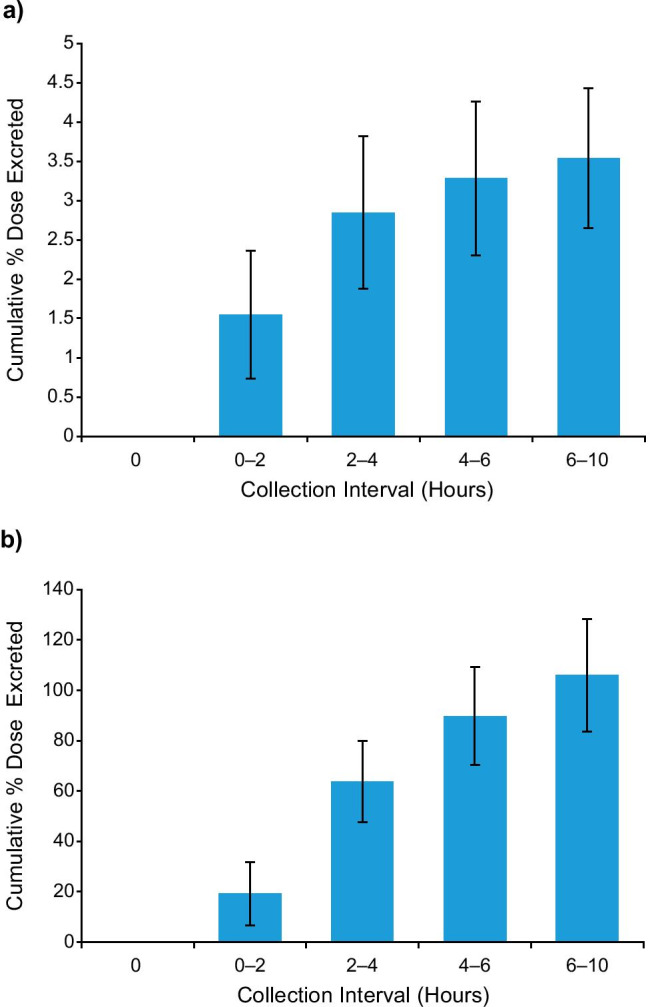
**a** Cumulative % dose of deferiprone and **b** deferiprone 3-O-glucuronide excreted following a 1500 mg dose of deferiprone

Urine samples were analyzed for the cumulative percentage of the dose excreted over 10 h as DFP (Fig. 2a) and as DFP-G (Fig. 2b). Excretion of unchanged DFP was 3.5 ± 0.9% (53.1 ± 13.4 mg), and 106 ± 22% (3598 ± 759 mg) was recovered in urine as DFP-G. The value of > 100% was likely due to the variability associated with manual methods of measuring urine volumes. The mean (SD) CLr for DFP was 20.9 (5.4) mL/min and 472 (178) mL/min for DFP-G.

### Safety assessments

Two patients (25%) reported a total of 5 adverse events (AEs) post-dose (abdominal pain in one subject; sore throat, headache, fatigue, and fever in the other subject). All AEs resolved by last contact, with the exception of sore throat and headache. AEs reported were of mild intensity and considered unrelated to DFP treatment. No hepatic abnormalities of clinical significance were noted at any point during the study, and no clinically significant laboratory results were observed. Moreover, there were no findings of clinical concern with regard to vital signs, physical examination, and 12-lead ECG.

## Discussion

In this phase I, open-label study of a single oral 1500 mg dose of DFP in subjects with SCD, the PK and safety profiles of DFP and its main metabolite, DFP-G, were characterized. Overall, DFP was rapidly absorbed and quantifiable in most subjects over the post-dose sampling period. Serum levels of both DFP and DFP-G rose steadily to maximum concentrations, and their respective peaks were followed by a steady decline. The majority of DFP was metabolized and excreted quickly and efficiently as DFP-G. Safety assessments of DFP in subjects with SCD included 2 patient reports of mild adverse events unrelated to treatment, and no indications of clinical concern were observed, indicating that a single oral 1500 mg dose of DFP was well tolerated by the subjects with SCD in this study.

Safe and effective chelation treatment options are important for patients with SCD. While iron chelators currently available have shown efficacy in the treatment of iron overload, some chelation therapy treatments are contraindicated in patients with hepatic or renal impairment [20, 21]. Previous studies in subjects with mild to moderate liver dysfunction reported that the disposition of DFP and DFP-G was generally similar among all subjects, regardless of degree of hepatic impairment [14, 22]. Similarly, previous studies comparing healthy volunteers with mild to severe renally impaired subjects demonstrated comparable drug exposure and found that no dosage adjustment of DFP is needed in patients with renal impairment [15]. These previous observations are notable because patients with SCD can experience up to a 68% increase in the prevalence of albuminuria with age [23, 24]. While subjects in the present study did not have significant renal impairment, it is a common complication associated with the progression of SCD [12, 24], and it limits patient options in chelation treatments. In the present study, the PK profile of DFP in adults with SCD is comparable to results observed previously in healthy volunteers and suggests that adaptation of the DFP drug dose is not necessary in SCD patients [22, 25]. Moreover, the long-term safety profile of DFP in a large number of patients with SCD and other transfusion-dependent anemias shows it is well tolerated with no new safety concerns [26]. While these findings are compelling, additional studies to investigate DFP in patients with SCD and renal impairment would also provide useful insight.

DFP is a small molecule with a low molecular weight compared with other commercially available iron chelators [27], and it has the ability to chelate excess intracellular iron from adversely affected organs (i.e., heart, liver, and endocrine tissues) without the likelihood of depleting normal intracellular iron levels [28]. The present study shows DFP has a relatively short half-life, and previous studies report that DFP-G (the main metabolite of DFP) does not have iron-chelating properties [27], suggesting that regular systemic exposure to DFP in the approved dose range is unlikely to put patients at risk for excessive iron chelation and unintended intracellular toxicity. The PK profile of DFP in SCD patients is similar to the PK profile in other iron-overload conditions [15], which provides valuable information regarding systemic exposure to DFP in SCD and potentially other related disease states. These unique characteristics of DFP may provide an additional benefit to patients with compromised liver function or renal impairment.

Recent epidemiology reports indicate that the mortality rates in adults with SCD have increased [13] and are attributed to chronic complications associated with the heart, lungs, and cerebrovascular system; acute infections; and renal disorders. Some of these chronic complications may be associated with iron buildup caused by frequent transfusions [29]. In contrast, mortality rates have been declining in children with SCD [13], likely due to modern advances in preventative medicine, such as prophylactic transfusion therapy. Thus, as individuals with SCD age, regular tests to monitor organ iron levels using accurate and well-tolerated methods [30] and access to adequate chelation treatment are becoming increasingly necessary to manage iron-related complications in patients with SCD treated with frequent blood transfusions.

While the findings from the present study are useful for understanding how DFP is metabolized in patients with SCD, the study design has limitations. Namely, the number of patients enrolled is relatively small. Future studies should include a larger patient population and more diverse demographics, including pediatric patients. In addition, the cumulative urinary excretion of DFP-G reported in this study was more than 100% of the administered doses (on a molar basis). It should be noted that standard bioanalytical methods for drug concentration measurement in urine can be associated with up to 15% variability [31]. Additionally, clinical sites with limited experience in the measurement technique might have inadvertently introduced variability, thereby causing over-estimation of the dose excreted. We also note that transfusional iron overload is often a chronic condition and the present study investigated only a single dose of DFP. However, single-dose and multiple-dose studies have been conducted in healthy volunteers and it was confirmed that there was no accumulation of DFP following multiple doses (unpublished data on file, Chiesi, USA).

In conclusion, the present study demonstrates that, similar to patients with thalassemia, DFP can be administered to patients with SCD without dose adjustments, as the drug and its main metabolite are safely and efficiently excreted in this patient population. Taken together, these findings highlight that DFP may address an unmet need for safe iron chelation in transfusion-dependent patients with SCD whose iron chelation options are currently limited.

## Supplementary Information

Below is the link to the electronic supplementary material.

Supplementary file1 (PDF 2093 KB) Online Resource 1: Study Timeline of Evaluations

## Data Availability

At this time, we will approve or deny data requests from external parties on a case-by-case basis. Chiesi reserves the right to deny requests for any and all legally appropriate reasons. Data requests that risk sharing participant-level data or proprietary information will not be approved.
