# Supplementary material for: The pharmacokinetic and safety profile of single-dose deferiprone in subjects with sickle cell disease
Source: Ann Hematol. 2022 Jan 4;101(3):533–9. doi: 10.1007/s00277-021-04728-0 (PMC8810455; doi:10.1007/s00277-021-04728-0)
Supplement: Supplementary file 1 — Supplementary file1 (PDF 2093 KB) Online Resource 1: Study Timeline of Evaluations [file 277_2021_4728_MOESM1_ESM.pdf]

Screening
Day -14 to -1

Study Procedure:
- Informed Consent
- Demography
- Medical History
- Prior & Concomitant Medication
- Physical Examination
- Height and Weight
- Vital Signs[1]
- Biochemistry
- Hematology
- Urinalysis
- Coagulation
- Virology
- Urine Drug/Alcohol Screen
- ECG (12-lead)
- Serum Pregnancy Test[2]
- Medical Events

Baseline
Day 0

Study Procedure:
- Prior & Concomitant Medication
- Physical Examination
- Height and Weight[3]
- Vital Signs[1]
- Biochemistry
- Hematology
- Urinalysis
- ECG (12-lead)
- Serum Pregnancy Test[2]
- Medical Events

Confinement Period
Day 1

Pre-dosing

Study Procedure:
- Prior & Concomitant Medication
- Vital Signs[1]
- Urine Drug/Alcohol Screen
- PK Blood Sampling[4]
- PK Urine Sampling[5]
- Medical Events

Dosing

Study Procedure:
Drug Administration: single 1500 mg (three 500 mg IR tablets) dose of FERRIPROX

Post-dosing

Study Procedure:
- Prior & Concomitant Medication
- PK Blood Sampling[4]
- PK Urine Sampling[5]
- Adverse Events

Checkout[6]

Study Procedure:
- Prior & Concomitant Medication
- Vital Signs[1]
- Biochemistry
- Hematology
- Urinalysis
- Coagulation
- ECG (12-lead)
- Adverse Events

Follow-up[7]
Day 7 ± 3

Study Procedure:
- Hematology
- Adverse Events

[1]Vital signs were evaluated at Screening, Baseline, Pre-dosing (up to 2 hours), and Checkout, or upon early withdrawal.

[2]Pregnancy test at Screening and Baseline was performed in women of childbearing potential. Follicle-stimulating hormone test was to be performed on postmenopausal women only.

[3]Weight only.

[4]PK blood samples were collected pre-dose and at 0.25 hours, 0.5 hours, 0.75 hours, 1 hour, 1.33 hours, 1.66 hours, 2 hours, 2.5 hours, 3 hours, 4 hours, 6 hours, 8 hours, and 10 hours post-dose.

[5]PK urine samples were collected at intervals of -2–0 hours pre-dose, and 0–2 hours, 2–4 hours, 4–6 hours, and 6–10 hours post-dose.

[6]Assessments were performed prior to release from the study center or upon early withdrawal.

[7]Patients returned to the site post-dose for the review of safety information and to provide a blood sample for the measurement of absolute neutrophil count.

ECG, electrocardiogram; IR, immediate release; PK, pharmacokinetic.
